# Electrical Capacitance versus Minirhizotron Technique: A Study of Root Dynamics in Wheat–Pea Intercrops

**DOI:** 10.3390/plants10101991

**Published:** 2021-09-23

**Authors:** Imre Cseresnyés, Bettina Kelemen, Tünde Takács, Anna Füzy, Ramóna Kovács, Mária Megyeri, István Parádi, Péter Mikó

**Affiliations:** 1 Centre for Agricultural Research, Institute for Soil Sciences, ELKH, Herman Ottó út 15, H-1022 Budapest, Hungary; cseresnyes.imre@atk.hu (I.C.); fuzy.anna@atk.hu (A.F.); kovacsrami@gmail.com (R.K.); istvan.paradi@atk.hu (I.P.); 2 Centre for Agricultural Research, Agricultural Institute, ELKH, Brunszvik u. 2, H-2462 Martonvásár, Hungary; megyeri.maria@atk.hu (M.M.); miko.peter@atk.hu (P.M.); 3 Department Plant Physiology and Molecular Plant Biology, Eötvös Loránd University, Pázmány Péter Stny. 1A, H-1117 Budapest, Hungary

**Keywords:** cereal–legume intercrops, drought stress, grain yield, in situ root methods, root growth

## Abstract

This study evaluated the concurrent application and the results of the root electrical capacitance (C_R_) and minirhizotron (MR) methods in the same plant populations. The container experiment involved three winter wheat cultivars, grown as sole crops or intercropped with winter pea under well-watered or drought-stressed conditions. The wheat root activity (characterized by C_R_) and the MR-based root length (RL) and root surface area (RSA) were monitored during the vegetation period, the flag leaf chlorophyll content was measured at flowering, and the wheat shoot dry mass (SDM) and grain yield (GY) were determined at maturity. C_R_, RL and RSA exhibited similar seasonal patterns with peaks around the flowering. The presence of pea reduced the maximum C_R_, RL and RSA. Drought significantly decreased C_R_, but increased the MR-based root size. Both intercropping and drought reduced wheat chlorophyll content, SDM and GY. The relative decrease caused by pea or drought in the maximum C_R_ was proportional to the rate of change in SDM or GY. Significant linear correlations (R^2^: 0.77–0.97) were found between C_R_ and RSA, with significantly smaller specific root capacitance (per unit RSA) for the drought-stress treatments. C_R_ measurements tend to predict root function and the accompanying effect on above-ground production and grain yield. The parallel application of the two in situ methods improves the evaluation of root dynamics and plant responses.

## 1. Introduction

Owing to methodological difficulties in assessing the growth and activity of intact root systems in the soil [1,2], the application and development of indirect, non-destructive techniques, including electrical methods, have received increasing attention in recent years [3,4].

The electrical capacitance of the root–soil system (C_R_), measured using a low-frequency alternating current (1 kHz AC) between one electrode fixed to the plant stem base and another inserted into the surrounding soil, was reported to correlate with the root system size (RSS) [5]. The first, generally accepted conceptual model, hypothesizes that roots are equivalent to leaky cylindrical capacitors, in which the electrically conductive root sap is separated from the conductive soil solution by polarizable membrane dielectrics [6]. Membranes store electric charges proportional to their surface area, modifying the phase and amplitude of the AC signal.

Nevertheless, some studies revealed the inconsistencies with Dalton’s proposals, and questioned the feasibility of the C_R_ method [7]. Dietrich et al. [8,9] suggested a revised model, and explored that C_R_ was dominated by the stem base between the plant electrode and the substrate surface (with a negligible contribution of the roots), and was correlated with a stem cross-sectional area. According to their explanation, the C_R_–RSS correlations are merely due to the allometric relationship between root and shoot traits. Another recent study also suggested that current leakage chiefly occurred in the root neck and proximal roots and much less in the distal segments [10]. In contrast, progressive root cutting and root immersion experiments in hydroponically grown plants supported that C_R_ is highly influenced by the roots located in the media [11]. There is a consensus that the current pathway inside the plant organs is influenced by tissue properties, e.g., suberization [4,6,12,13]. Therefore, one advantage of the capacitance method is that C_R_ is a reflection of root size and physiological status, i.e., water content, membrane integrity and permeability, and endodermal maturation, driven by ontogenic changes and environmental (stress) factors [13]. Due to the allometry between the proximal root regions that mostly contribute to current flow and the distal fine roots responsible for solute absorption [8,9,10], C_R_ is related to the water uptake activity of the root system (referred to as “root activity”) as a whole [6,14,15].

The rapid C_R_ method is appropriate for monitoring the same plant over time, and for screening large plant populations [16]. As the neighboring plants (with intermingling roots) are not connected electrically, C_R_ provides plant-scale information [13]. An evident drawback to using the technique is the complete lack of root visualization. It is difficult to quantify root traits from capacitance data, which are more suitable for the relative comparison of RSS for the same species, grown in the same substrate and measured under the same conditions [16]. Although C_R_ is strongly affected by the soil water content (SWC), this can be taken into account with species-specific functions [15]. The soil dielectric response tends to reduce the efficiency of the method, particularly in the case of complex soils abounding in surface-charged colloidal particles [3]. Nevertheless, despite the uncertainties about the underlying biophysical mechanisms, the method has proven useful in several studies to evaluate RSS in herbaceous native and crop species and in tree saplings grown in pots [14,17,18,19] or in the field [13,15,16,20].

The minirhizotron (MR), a more widely used non-destructive visualization technique, allows the dynamic study of root traits at particular locations in the soil profile, including root architecture, branching, depth distribution, length density, production, longevity, mortality, turnover and decomposition [2,21]. However, MR represents only part of the root system, with limited resolution for the fine roots that are responsible for water uptake [22]. The main biases are attributed to the artificial MR tube surface and the poor soil-tube wall contact (including soil gaps), which may result in modified temperature conditions, altered water flow and decreased soil penetration resistance, potentially changing the observed root density and traits [7,23]. The critical points in the MR technique are the assessment of root physiological status (activity) by visual evaluation (e.g., color, shrinking, contour smoothing, blotting), and the differentiation between the roots of individual plants [21]. The available data are often limited due to the time-consuming image analysis, and to the lack of automatic image processing methods that give satisfactory results for MR pictures [2,24].

We surmised that the use of the entirely different C_R_ and MR methods in the same root study would be beneficial. In the framework of an EU H2020 project, the aim was to use the two techniques concurrently (which has not previously been reported) in intercropping systems, which are increasingly important in sustainable agriculture [25,26]. The more efficient resource utilization in species mixtures compared to sole crops may result in benefits such as higher yield equivalent ratios and yield stability, improved grain quality, enhanced lodging resistance, improved soil conservation and better control of pests and weeds [27,28]. Cereal–legume, including wheat–pea mixtures, are the dominant intercrops in organic farming in Europe [26,29]. Symbiotic N_2_ fixation in pea more or less satisfies the high N demand of the cereal [25,30]. The complementary resource use and, therefore, growth and yield components, highly depend on the root dynamics, and the root architectural modifications of the mixed species [1,31]. The wheat root response to intercropping under altered conditions is often highly variable and is hardly predictable [32]. Therefore, in situ root investigation methods were deemed appropriate to track root development over time in mixed crop culture, and to evaluate the responsiveness of the selected wheat cultivars to intercropping under different irrigation conditions. Winter wheat and pea mixtures, grown in containers in the greenhouse, were used for the present methodological study. Although this controlled environment was likely not representative of field conditions, it allowed us to standardize experimental parameters, to adjust contrasting soil moisture regimes, and to eliminate weather anomalies.

In this study, C_R_ and MR measurements were performed in the same intercrop system, aiming specifically (i) to analyze and compare the results provided by the methods during monitoring the response of root dynamics of various wheat genotypes to intercropping and drought, and (ii) to evaluate the potential benefits of the simultaneous application of the two approaches.

## 2. Results

### 2.1. Root Electrical Capacitance

**Figure 1:**
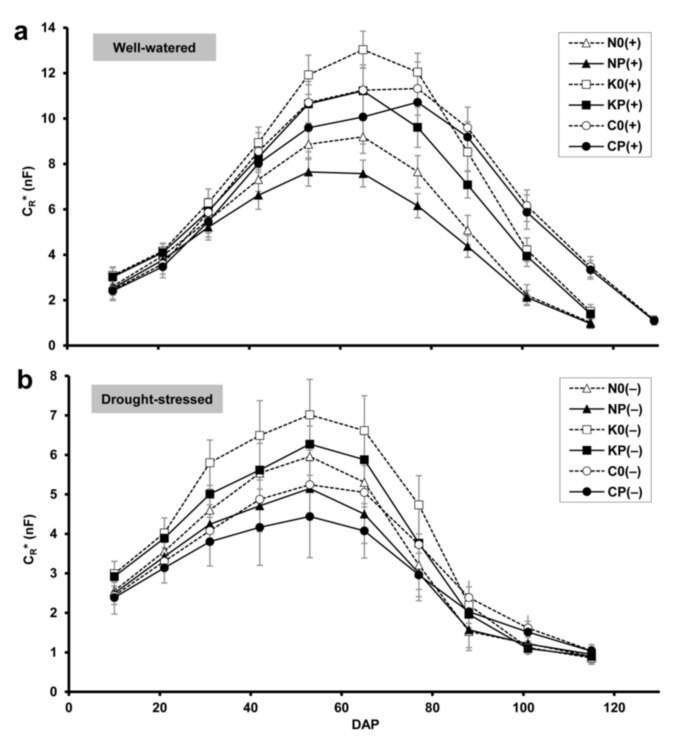
Changes in the apparent root electrical capacitance (C_R_*; in nanofarads, nF) of wheat with plant age (DAP: days after planting) under (**a**) well-watered and (**b**) drought-stressed conditions. Bars show standard deviations (*n* = 30). Treatment codes: N: wheat cv. Mv Nádor; K: cv. Mv Kolompos; C: YQCCP population; 0: wheat sole crop; P: wheat–pea intercrop; (+): well-watered; (–): drought-stressed.

The apparent root capacitance (C_R_*) obtained for the well-watered sole wheat crops increased rapidly over time until DAP 65 for cultivars ‘Mv Nádor’ (9.2 nF) and ‘Mv Kolompos’ (13.0 nF), and until DAP 77 for YQCCP (11.3 nF; Figure 1a). The peak of C_R_* coincided with the early or full flowering stage (BBCH 61–65) in each case. The intercropped wheats showed similar phenology and temporal C_R_* patterns. The presence of pea caused a decrease in wheat root capacitance (with maxima of 7.6, 11.2 and 10.7 nF for ‘Mv Nádor’, ‘Mv Kolompos’ and YQCCP, respectively); in general, this effect was significant from DAP 31 (stem elongation) to DAP 88 (milky or early dough) (Table S1).

Under drought, both sole and intercropped wheat exhibited a moderate increase in C_R_* over time, with peak values ranging from 4.4 to 7.0 nF on DAP 53 (boot stage; BBCH 41–49) for the various cultivars (Figure 1b). Wheat phenology was accelerated by drought, especially for YQCCP, which flowered two weeks earlier. Drought provoked a sudden decrease in C_R_* after flowering (between DAP 65 and 88) in all treatments. As in the case of the well-watered treatment, the pea intercrop significantly reduced wheat root capacitance under drought during the greater part of the growing season (Table S1). In each treatment, drought resulted in a significant decrease in C_R_* from DAP 21 (leaf development stage; BBCH 14–15) onwards (Table S2).

### 2.2. Minirhizotron Image Analysis

**Figure 2:**
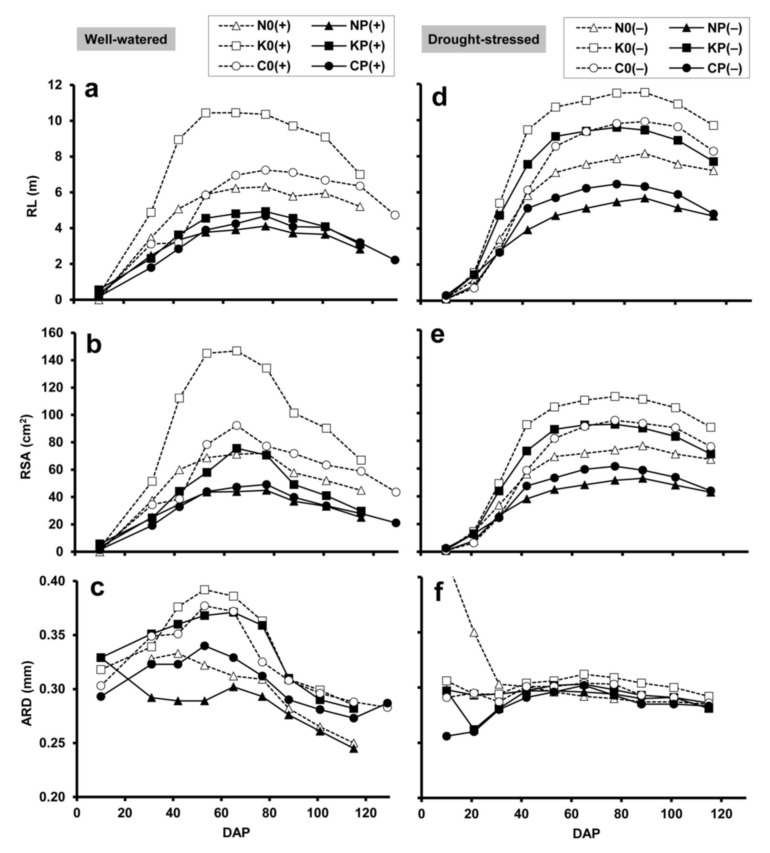
(**a**,**d**) Root length (RL), (**b**,**e**) root surface area (RSA) and (**c**,**f**) average root diameter (ARD), obtained by minirhizotron image analysis, in relation to plant age (DAP: days after planting) under well-watered (**a**–**c**) and drought-stressed (**d**–**f**) conditions. RL and RSA are sums from the three soil depths (20, 50 and 80 cm), ARD is a weighted average. For treatment codes, see Figure 1.

MR image analysis demonstrated that the total RL and RSA (at 20–80 cm depths) of the well-watered crops increased rapidly until the flowering period (DAP 65–77), and subsequently declined to maturity (Figure 2a,b). In both the sole and mixed crops, the root system proved to be relatively small, medium and large, respectively for ‘Mv Nádor’, YQCCP and ‘Mv Kolompos’. According to the paired *t* test, maxima of RL and RSA were significantly (*p* < 0.01) higher in the sole wheat culture compared to the corresponding mixture. ARD showed similar temporal patterns, reaching a maximum between DAP 42 (late stem elongation; BBCH 34–36) and DAP 65 (early flowering), and gradually decreased thereafter (Figure 2c). Wheat genotypes ‘Mv Nádor’, YQCCP and ‘Mv Kolompos’ were characterized by thin, medium and thick root systems, respectively. In the pure wheat stands ARD significantly (*p* < 0.01) exceeded that of the intercrop counterparts.

Under drought conditions, there was a prolonged increment in total RL and RSA until DAP 77 or 88 (milky or dough stages; BBCH 75–85), followed by a slight decline during maturity (Figure 2d,e). Apart from this, the differences observed in these root traits between the wheat types and between the sole and intercrops were similar to those obtained for the corresponding well-watered treatments. In most cases, water deficit increased the maximum RL and RSA, the only exception being the ‘Mv Kolompos’ sole crop, in which RSA was reduced by drought (however, this could be due to the relatively high basis of comparison with the well-watered plants). The paired *t* test showed that the maximum of RL was significantly (*p* < 0.01) increased by drought. The peak value of ARD was significantly (*p* < 0.05) reduced both due to intercropping and due to drought, and was found to be less variable between the treatments and over time under drought conditions (Figure 2f).

**Figure 3:**
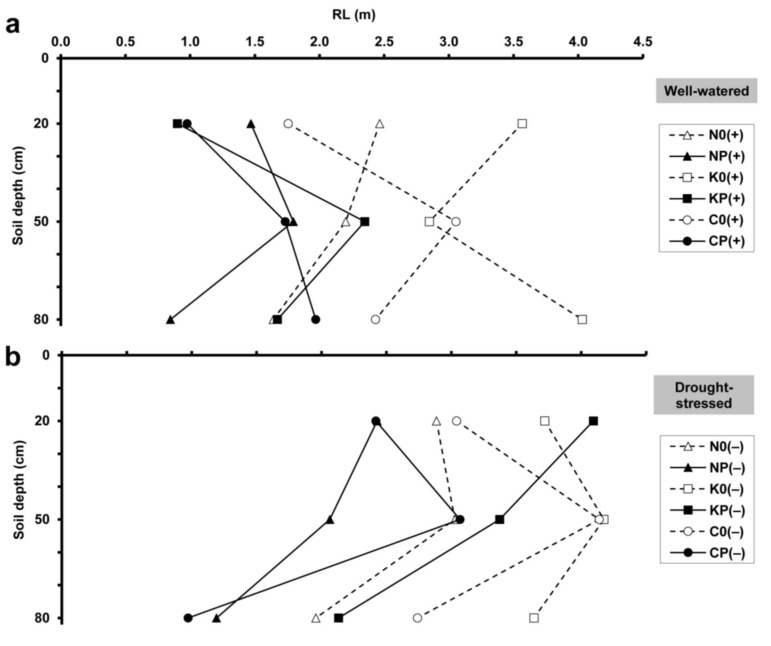
Maximum root length (RL) at 20, 50 and 80 cm soil depths, obtained by minirhizotron image analysis, under (**a**) well-watered and (**b**) drought-stressed conditions. For treatment codes, see Figure 1.

The well-watered wheat cultivars showed different RL distributions in the soil profile (Figure 3a), while in dry soil all three wheats exhibited the highest RL at the 50 cm depth (Figure 3b). RL was usually reduced by intercropping with pea and was increased by drought at each depth.

### 2.3. Chlorophyll Content and Yield Components

**Figure 4:**
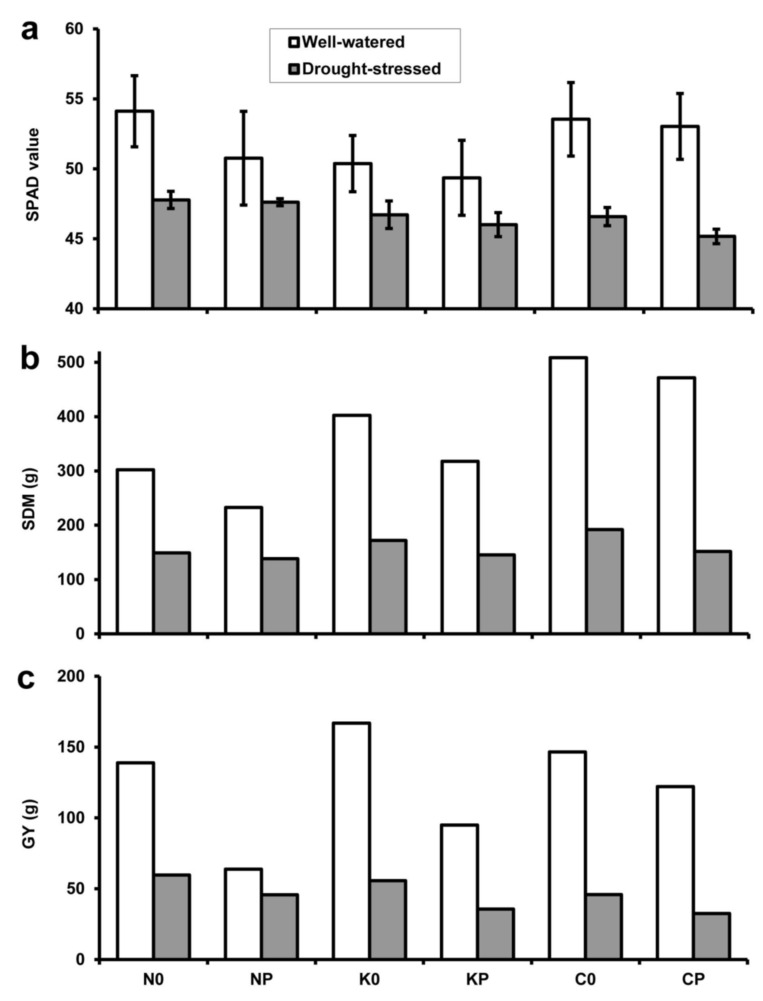
(**a**) Leaf chlorophyll content as SPAD value (mean ± SD; *n* = 15), (**b**) total shoot dry mass (SDM) and (**c**) total grain yield (GY) under well-watered (white columns) and drought-stressed (grey columns) conditions. For treatment codes, see Figure 1.

The SPAD values measured for wheat leaves ranged from 45.2 ± 0.5 to 54.1 ± 2.5 (mean ± SD; *n* = 15; Figure 4a). The presence of pea led to an overall decrease in chlorophyll content, but the change was only significant in the case of well-watered ‘Mv Nádor’ (*p* < 0.01), drought-stressed ‘Mv Kolompos’ (*p* < 0.05) and YQCCP (*p* < 0.001). Water deficit significantly (*p* < 0.01) reduced the SPAD value in each treatment.

Total wheat SDM per treatment was found to be between 138 and 508 g (Figure 4b). When wheat genotypes grown under the same experimental conditions were compared, SDM was always relatively low, medium and high for ‘Mv Nádor’, ‘Mv Kolompos’ and YQCCP, respectively. According to the paired *t* test, SDM was reduced both by intercropping (*p* < 0.05) and particularly by drought stress (*p* < 0.01).

Total wheat GY varied between 36 and 167 g in the treatments (Figure 4c). Unlike SDM, sole-cropped ‘Mv Kolompos’ had the highest GY under well-watered conditions, and in the case of drought, ‘Mv Nádor’ exhibited the highest and YQCCP the lowest GY in both sole and mixed stands. The effect of pea intercropping and drought on GY was significant at the *p* < 0.05 and *p* < 0.01 levels, respectively.

### 2.4. Relative Changes in Plant Parameters

**Figure 5:**
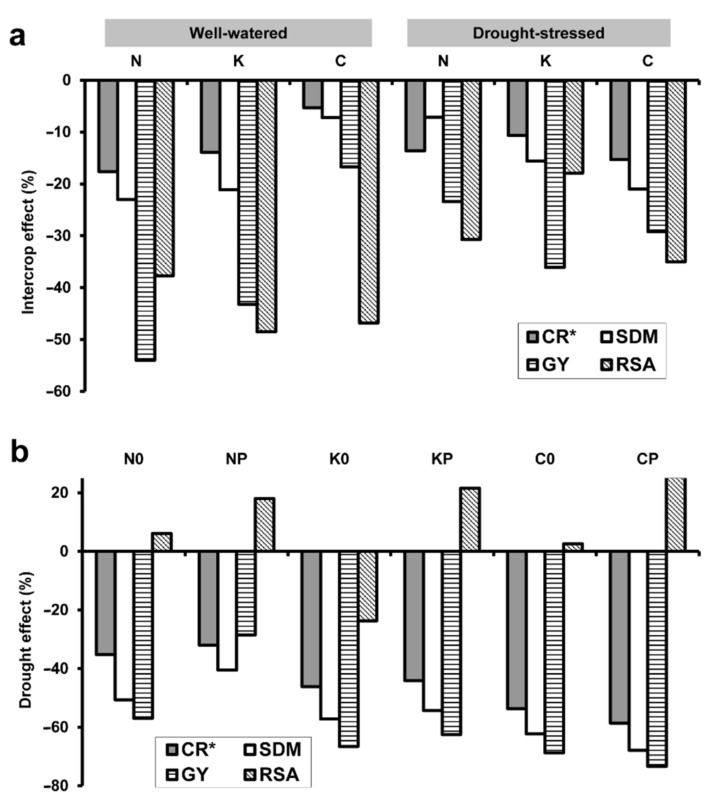
Percentage changes in the apparent root electrical capacitance (C_R_*), total shoot dry mass (SDM) and total grain yield (GY) of wheat and in the root surface area (RSA) caused by (**a**) pea intercropping and (**b**) drought stress. In the case of C_R_* and RSA, maximum values (detected at the wheat flowering stage) were used for calculation. For treatment codes, see Figure 1.

The percentage changes in maximum C_R_*, SDM, GY and maximum RSA in response to pea intercropping and drought were calculated for each wheat genotype. In the case of well-watered plants, the relative decrease in C_R_*, SDM and GY caused by pea intercropping was the lowest (5%, 7% and 17%, respectively) for YQCCP, and the highest (18%, 23% and 54%) for ‘Mv Nádor’ (Figure 5a). Conversely, RSA showed a considerable reduction for ‘Mv Kolompos’ (49%) and the composite population (47%), and a relatively moderate decrease for ‘Mv Nádor’ (38%).

The presence of pea resulted in a similar decrease (11–15%) in C_R_* for all the cultivars when grown under drought conditions (Figure 5a). SDM, GY and RSA dropped by 7–21%, 23–36% and 18–35%, respectively, compared to the sole crops. Pea caused the highest relative changes in C_R_*, SDM and RSA in the YQCCP population, whereas the greatest loss in GY was observed for the cultivar ‘Mv Kolompos’.

Drought stress reduced C_R_* by 32–59%, SDM by 41–68% and GY by 29–73% (Figure 5b). In both sole and intercrops, the relative changes in all three parameters were comparatively low, medium and high for genotypes ‘Mv Nádor’, ‘Mv Kolompos’ and YQCCP, respectively. Except for the ‘Mv Kolompos’ sole crop, RSA was increased by drought in all cases (by 3–25%), but more intensely in the intercrops.

### 2.5. Relationship of Electrical Capacitance to Root Surface Area

**Figure 6:**
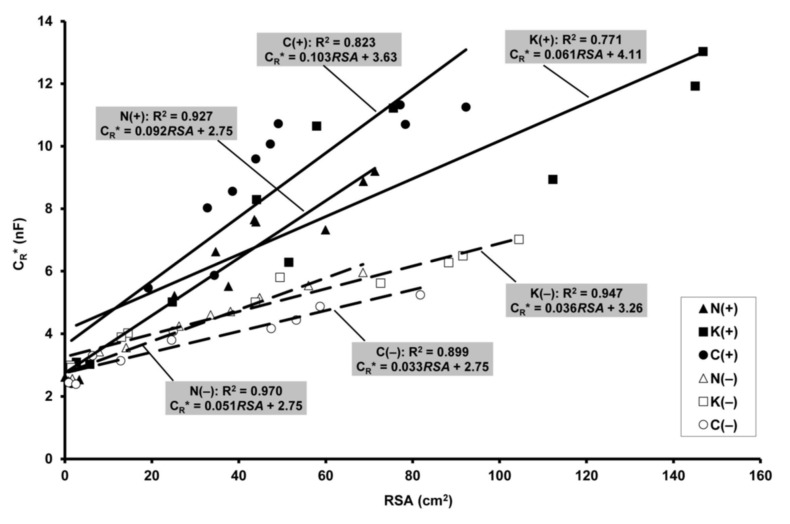
Linear relationships between the apparent root electrical capacitance (C_R_*) and root surface area (RSA) in well-watered (filled symbols and solid lines) and drought-stressed (empty symbols and dashed lines) treatments. Only data obtained up to the flowering stage of the wheat genotypes were considered, and were pooled over the two pea treatments. For treatment codes, see Figure 1.

Highly significant positive linear correlations (F: 26.9–255; R^2^: 0.771–0.970; *p* < 0.001) were found between C_R_* and RSA for each wheat genotype and water supply (Figure 6). Analysis of covariance showed significant differences in regression slope between the genotypes under both well-watered (F_2,26_ = 6.46; *p* < 0.01) and drought-stressed conditions (F_2,24_ = 5.08; *p* < 0.05). Drought decreased the slope significantly for ‘Mv Nádor’ (F_1,16_ = 25.3; *p* < 0.01) and YQCCP (F_1,18_ = 15.8; *p* < 0.01), but non-significantly for ‘Mv Kolompos’ (F_1,16_ = 3.00; *p* > 0.05).

## 3. Discussion

### 3.1. Root Dynamics

Both C_R_* and the MR-based root properties were found to exhibit strong patterns depending on growth status, peaking at wheat flowering for the well-watered treatments. In the case of water deficit, C_R_* had already started to decrease at booting, whereas the peaks of RL and RSA were postponed to the milky or dough stages. These findings are consistent with data in the literature obtained using various root methods. Annuals translocate and assimilate preferentially to the aboveground parts after flowering, and RSS increases slightly or even declines as the roots decay [33]. Previous studies reported maximum wheat root biomass, root length and root activity (water use) at about anthesis [34,35], with strong links to the concurrent peaks of green leaf area and whole-plant transpiration [36]. However, the patterns of wheat root growth may be less determinate [37], as was indicated in the present experiment by the C_R_* and RL data obtained for the composite cross population YQCCP, which was genetically and phenologically heterogeneous [38]. Wheat ARD was reported to peak during the flowering stages, and was found to be smaller in dry soil [22].

### 3.2. Effect of Drought and Intercropping

Drought stress shortened the wheat vegetation period. Root activity (C_R_*) began to decline before flowering, and showed a sudden decrease thereafter, due to enhanced lignification and accelerated root senescence [13,39]. Nevertheless, the decreasing trend in RL and RSA was observed later under drought. This was reported due to the delayed disappearance of dead roots (the visual determination of when roots reach the dead stage is subjective), especially in dry soil, where decomposition is slower [40].

The C_R_ and MR methods indicated that the presence of pea had a negative effect on root growth parameters. Although intercropping systems generally give a higher total yield compared to sole crops (higher land equivalent ratio), the single component species often responds to intercropping with a reduction in biomass and grain yield [41]. The high plant density in additive intercrops often leads to strong interspecific competition between wheat and legumes below ground for soil nutrients and water (principally under drought), and above ground for light [41,42]. It was reported that pea cultivars with vigorous early development and climbing growth habit tended to overgrow the wheat (which provided mechanical support), limiting light interception for the cereal canopy [43]. This was clearly observed in the present study: pea overtopped the wheat, and tied up the wheat leaves with tendrils during the vegetative phase. Pea significantly reduced the SPAD chlorophyll content in well-watered ‘Mv Nádor’ and in drought-stressed ‘Mv Kolompos’ and YQCCP, in which greater aboveground biomass loss was detected under the given experimental conditions. The decreased chlorophyll content of flag leaf (the major source leaf) by drought indicated an enhanced senescence and nutrient remobilization, leading to reduced shoot and root productivity and grain yield [44,45]. ‘Mv Nádor’ and YQCCP were the least and the most tolerant to intercropping, respectively, under well-watered conditions, in contrast with the tolerance under drought. The reduction in cereal yield by the competitive interactions may be overcome by reducing the intercrop density.

### 3.3. Benefits and Drawbacks of the Combined Approach of C_R_ and MR

The two different root methods provided apparently conflicting results, in that drought stress substantially decreased maximum C_R_* (by 32–59%), but increased RL and RSA. Increase in root mass is a common response to limited water supply, but plant responses to drought depend greatly on timing, duration and severity [39]. In the present case, the drought was relatively moderate during the early growth stages, but became severe during flowering and grain filling, which are the most sensitive periods of wheat growth [33]. Mild drought in early growth stages have been reported not to inhibit or even to stimulate wheat root production [46], as was indicated by the present MR investigation. In accordance with the current C_R_* data, severe drought was observed to reduce root activity by reducing the uptake of water and promoting tissue maturation [39]. Nevertheless, another possible explanation for the decreased C_R_* is the smaller shoot growth and stem diameter, altering the allometry between root and shoot [8,9]. This should be taken into account as well, considering the distinct depths of MR and C_R_ measurements, and the uncertainties about the relative influence of deep vs. shallow roots on C_R_* [2,10].

An enhanced root/shoot ratio under drought is considered a typical response of plants, including wheat [46], to water deficit. In relative terms, the restricted aboveground growth (associated with reduced leaf area and total plant transpiration) decreased the water uptake of the whole root system, indicated by the decline in C_R_*. Comparing the wheat cultivars studied, the relative change in maximum C_R_* induced by drought (like the effect of pea intercropping) was proportional to that in SDM and GY. ‘Mv Nádor’ was found to be the most tolerant to drought both in the sole crop and the intercrop, while the YQCCP was the most sensitive. Drought-stressed plants exhibited lower specific root capacitance (per unit RSA), manifested in the smaller slopes of the C_R_*–RSA regression lines. On the one hand, this finding may support the functional aspect of the C_R_ method, pointing out that C_R_ represents not only the geometrical size but also the physiological status (e.g., tissue maturity, root decay) of the root system. On the other hand, however, it demonstrates the limited comparison ability of capacitance data collected under contrasting growth conditions due to their different relationships with real RSS values. It is notable that the MR data indicated a larger RSS under drought, while the C_R_ measurements did not show such a trend at all.

Many previous studies demonstrated that MR-based RL and RSA data measured at anthesis in winter wheat (mainly in deeper soil layers) were positively correlated with plant water use and grain yield [24,33,47]. However, this was not the case in the present experiment, where well-watered and severely drought-stressed plants were compared. C_R_* proved to be a more sensitive indicator of SDM and GY losses. Nevertheless, if the capacitance method is applied alone, it remains unclear to what extent the change in root size or root activity, or even only the altered root to shoot ratio (reduced stem cross-sectional area), affects C_R_*. Transformation of measured C_R_ into C_R_* using predetermined specific calibrations is only required to monitor root dynamics in field-grown plants under variable SWC, but is unnecessary for single measurements (which should be made around anthesis) performed under spatially homogeneous moisture conditions [16]. However, caution is required when applying the capacitance technique to compare cultivars, considering that, as verified in the current study, the relationship between C_R_* and RSS may differ markedly. Furthermore, drought-induced changes in root membrane thickness and root depth distribution (including the death of shallow fine roots and the proliferation of deep roots) can be expected to weaken the capacitance response [10,13].

### 3.4. Conclusions

The current research confirmed that the C_R_ and MR measurements reveal different characteristics of the root system. The parallel use of the two methods in the same plant–soil system is able to give more information about the seasonal pattern of root growth and function, and principally about the influence of experimental conditions on root traits. Root capacitance predicts the accompanying effects on aboveground biomass production and grain yield in a comparative study, whereas the MR method is more suitable for quantifying relevant RSS variables. The concurrent application of the two non-destructive techniques could provide a better understanding of how various crop genotypes respond to different cultivation practices (e.g., tillage, sowing density, fertilization) or environmental conditions (e.g., drought, elevated CO_2_, nutrient deficiencies). The dual methodology could be particularly beneficial under contrasting growing conditions (when root and shoot development is often asynchronous), and in plant mixtures, in which the roots of the component species are difficult to distinguish with the MR method. Nevertheless, considering the lack of treatment replications in the present model system, more extensive studies are needed for better evaluation of the benefits and drawbacks of using both techniques combined, and to fill the gap of knowledge about the disparity of changes in C_R_ and MR data under stress.

## 4. Materials and Methods

### 4.1. Plant Material and Growth Conditions

The experimental design was factorial with (1) three winter wheat (*Triticum aestivum* L.) genotypes: the cultivars ‘Mv Nádor’ (“N”) and ‘Mv Kolompos’ (“K”) and the YQCCP composite cross population (“C”); (2) two cropping systems: wheat as sole crop (“0”) and a wheat–pea (*Pisum sativum* L., cv. Aviron) intercrop (“P”); and (3) two water treatments: well-watered (“+”) and drought-stressed (“–“). ‘Mv Nádor’ is an intensive, short (60–80 cm), early maturing variety, whereas ‘Mv Kolompos’ is a robust, less intensive, medium-tall (85–95 cm), medium-early cultivar. The late maturing composite cross population was created by crossing 20 parental lines in the Organic Research Centre, UK [38]. The wheat cultivars selected for the experiment were examined as a model genotypes of conventional (‘Mv Nádor’) and organic (‘Mv Kolompos’) farming systems, while the population (YQCCP) was used as a model to examine the effects of an organic heterogeneous material compared to the homogeneous varieties. The winter pea ‘Aviron’ is a determinate, semi-leaflet, medium-early, medium-tall (70–85 cm) cultivar with rapid early growth.

Six 1000 L cubic plastic containers were used, two (a well-watered and a drought-stressed) for each wheat genotype (Figure S1). Each container was divided into two parts (with 0.5 m^2^ surface area) by placing a 2 cm thick plastic sheet vertically, to separate the sole crop of a given genotype from the corresponding intercrop. Perpendicular to the sheet, three clear polycarbonate MR tubes (110 cm long, 70 mm outer diameter, 4 mm wall thickness) with end caps were installed horizontally through each container at depths of 20, 50 and 80 cm below the soil surface [21]. The containers were filled with topsoil (0–30 cm) taken from the nearby certified organic field (N 47°18′41″, E 18°46′48″). The soil was a haplic chernozem according to the FAO-WRB [48] classification, which consisted of 36.2% sand, 40.7% silt and 23.1% clay, with a pH_H2O_ of 7.61, 16.3 mmol 100 g^–1^ cation exchange capacity, 1.70% CaCO_3_, 3.36% humus content, 1822/364/459 mg kg^–1^ N/P/K content, and 1.42 g cm^–3^ bulk density. The SWC values at saturation, field capacity and wilting point were 47.9%, 28.9% and 8.7%, respectively (the soil water retention curve was determined with a pressure membrane apparatus). The soil packed into the three well-watered containers was at around field capacity, but for the three drought-stressed containers the soil was first spread out to dry to approx. half of field capacity. The bottom of the containers was filled with fist-size stones covered with agro-textile to ensure drainage. Large clods were broken up, organic debris was removed, and the soil was compacted carefully during filling (to avoid injuring the MR tubes). SWC at 20 and 50 cm soil depths was recorded every 2 h in each experimental unit with Decagon EC-5 sensors (Decagon Devices Inc., Pullman, WA, USA) connected to Em5b dataloggers.

Wheat seeds were germinated in moistened 30 mm Jiffy peat pellets (Jiffy Int. AS, Kristiansand, Norway) at 22 °C for 10 days, after which the seedlings were vernalized for 6 weeks at +4 °C, 10/14 h (light/dark). Ten days before the end of vernalization, pea seeds were germinated in the same way and were planted in the containers concurrently with the wheat. Each container held a sole crop of a genotype on the one side, and the intercrop of the same genotype on the other side. Each treatment (occupied a half container) consisted of 4 rows of 38 wheat plants with 12.5 cm row spacing (0.5 × 1 m; ~300 plants m^–2^), perpendicular to the MR tubes. Intercropping was additive, with 25 pea plants (50 plants m^–2^), five in each wheat interrow and longitudinal edge. The containers were maintained in a tempered greenhouse, initially at 15/10 °C for 12/12 h (light/dark), changing gradually to 23/18 °C for 16/8 h during the plant growth period. The containers were arranged in two rows according to the irrigation regime, with different orders for the genotypes within a row. The orientation ensured the uniform light and ventilation conditions for each container. The SWC in the three well-watered containers was reduced to 60–70% field capacity (17–20 *v/v*%), and then kept at this level by weekly irrigation. The three drought-stressed containers were given a little water (5 mm) at planting, after which the soil was allowed to dry to near wilting point (~9 *v/v*%), which was maintained by slight (6–10 mm) irrigations thereafter.

In the well-watered containers, the SWC at 20 and 50 cm depths decreased from field capacity to 17–20 *v/v*% by approx. DAP (days after planting) 70 (Figure S2), and varied between 18 and 25 *v/v*% in the 0–12 cm layer on the days when C_R_ measurements were made. In the case of drought stress, the soil moisture approached the wilting point at 20 and 50 cm by DAP 50 (wheat booting stage) and DAP 70 (end of flowering), respectively. A range from 9 to 12 *v/v*% SWC values were detected at 0–12 cm during the C_R_ measurements.

### 4.2. Electrical Capacitance Measurements

Electrical measurements were performed on ten occasions throughout the growing season, between DAP 10 and 115, and an eleventh measurement was made on DAP 129 for the well-watered composite wheat owing to its longer growth period. Thirty wheat plants were randomly selected from the inner rows of each treatment. The SWC at 0–12 cm (equal to the insertion depth of the C_R_ ground electrode) was recorded in the root zone of each individual plant with a pre-calibrated Campbell CS620 handheld TDR meter (Campbell Sci. Ltd., Loughborough, UK). Volumetric SWC values were converted to relative water saturation (*θ*_rel_) on the basis of the predefined saturation water content. Thereafter, parallel C_R_ was measured for each selected plant with an Agilent U1733C portable LCR instrument (Agilent Co. Ltd., Penang, Malaysia) at 1 kHz, 1 V AC. The ground electrode was a stainless steel rod, 15 cm long and 6 mm in diameter (303S31; RS Pro GmbH, Gmünd, Austria), pushed vertically into the soil 5 cm from the stem to a depths of 12 cm. The plant electrode was clamped to a 4 mm wide strip cut from a 25 µm thick aluminum foil. The strip was smeared with conductivity gel, and was tied around all the basal parts of the plant 15 mm above the soil surface [20].

In order to take the spatial and temporal variability in SWC into account, all the C_R_ data were converted into an apparent (saturation) capacitance, C_R_*, according to the species-specific function: C_R_* = C_R_·5.807e^–1.775*θ*rel^, using the *θ*_rel_ values associated with the relevant C_R_. A detailed description of how the empirical equations were calculated can be found in Cseresnyés et al. [15] C_R_*, which is equivalent in practice to the capacitance measured in a water-saturated soil (*θ*_rel_ = 1), was considered as a proxy of the functional root extent (root activity). The wheat growth stage was determined on each measurement day using the BBCH scale [49].

### 4.3. Minirhizotron Technique

Only one MR tube per soil depth was installed in each treatment because of the relatively small container volume. MR was primarily used to track the root seasonal patterns and to evaluate the effect of intercropping and drought. On the same days when C_R_ was detected, root images (21.6 × 19.6 cm; 300 dpi) were recorded for each treatment and depth using a CI-600 rotary scanner (CID Bio-Science Inc., Camas, WA, USA) set to the same position in the MR tube throughout the experiment. No images were taken in the well-watered containers on DAP 21 due to a technical problem.

The images were processed with *RootSnap!* software (ver. 1.3.2.25), which provided root length (RL), root surface area (RSA) and average root diameter (ARD). As it was impossible to distinguish between wheat and pea roots, the total root pool of the component species was evaluated. Nevertheless, wheat roots were considered to be dominant due to the much higher planting density and the dense adventitious root system [50]. Only active roots, identified by their white to pale brown color, were considered, whereas dark brown, blurry roots were disregarded [21].

### 4.4. Leaf Chlorophyll Content and Post-Harvest Measurements

The flag leaf chlorophyll content was estimated in situ at anthesis to characterize the physiological status of wheat grown under different conditions. Fifteen plants from the central rows of each treatment were investigated using a Minolta SPAD-502 m (Konica Minolta Inc., Osaka, Japan). Three measurements taken on the same plant were averaged.

At wheat maturity (on DAP 130 and 119 for the well-watered and drought-stressed treatments, respectively), the two crops were hand harvested separately just above the soil surface. The wheat biomass was oven-dried at 70 °C to constant weight to determine the total shoot dry mass (SDM), after which the ears were threshed manually to obtain the total grain yield (GY).

### 4.5. Data Analysis

The data were analyzed using Statistica software (ver. 13; StatSoft Inc., Tulsa, OK, USA). The effect of pea intercropping and drought on the C_R_* and SPAD values was evaluated with an unpaired *t* test (*p* < 0.05). Welch’s correction was applied when the *F* test indicated significant differences between the variances. A paired *t* test was used to analyze the influence of intercropping and drought on the maxima of RL, RSA and ARD, and on SDM and GY (in this case, treatments differing only in cropping design or in water regime were paired). Simple linear regression was performed to relate C_R_* to RSA. In this case, only the data collected until the wheat flowering stage (when C_R_* and RSA peaked) were used, and pooled over cultivar sole and intercrop. The reason for this is that the correlation between capacitance and root size properties is much stronger during the most active (vegetative to flowering) plant growth stages characterized by intensive root growth, and becomes weaker during maturity stages, when root suberization, reduced activity and death can result in a sudden decrease in capacitance [6,10]. The regression slopes were compared with analysis of covariance.

## Data Availability

Not applicable.

## Supplementary Materials

The following are available online at www.mdpi.com/article/10.3390/plants10101991/s1. Table S1. Effect of pea intercropping on the apparent root electrical capacitance (CR*) of wheat at different plant ages (DAP: days after planting). Unpaired *t* test (*n* = 30) or Welch’s test [superscript (W) in the table] was used, based on the homogeneity of variances. NS: non-significant; * *p* < 0.05; ** *p* < 0.01; *** *p* < 0.001. Treatment codes: N: wheat cv. Mv Nádor; K: cv. Mv Kolompos; C: YQCCP population; 0: wheat sole crop; P: wheat–pea intercrop; (+): well-watered; (–): drought-stressed. Table S2. Effect of drought stress on the apparent root electrical capacitance (CR*) of wheat at different plant ages (DAP: days after planting). Unpaired *t* test (*n* = 30) or Welch’s test [superscript (W) in the table] was used, based on the homogeneity of variances. NS: non-significant; * *p* < 0.05; ** *p* < 0.01; *** *p* < 0.001. For treatments codes, see Table S1. Figure S1: Schematic for the experimental design. Treatment codes: N: wheat cv. Mv Nádor; K: cv. Mv Kolompos; C: YQCCP population; 0: wheat sole crop; P: wheat–pea intercrop; (+): well-watered; (–): drought-stressed. Figure S2: Changes in volumetric soil water content (SWC) with plant age (DAP: days after planting) in well-watered [(+)] and drought-stressed [(–)] treatments at different soil depths. SWC was recorded continuously at 20 and 50 cm depths using automatic data loggers, but only concurrently with root electrical capacitance measurements in the 0–12 cm layer with a handheld TDR meter. Data were averaged across the three wheat cultivars and two pea treatments. Horizontal dashed lines indicate water content at field capacity (FC; 0.02 MPa) and wilting point (WP; 1.5 MPa).

## Abbreviations

AC	Alternating current
ARD	Average root diameter
C_R_	Root electrical capacitance
C_R_*	Apparent (saturation) root electrical capacitance
DAP	Days after planting
GY	Grain yield
MR	Minirhizotron
RL	Root length
RSA	Root surface area
RSS	Root system size
SDM	Shoot dry mass
SWC	Soil water content
