# Supplementary material for: Electrical Capacitance versus Minirhizotron Technique: A Study of Root Dynamics in Wheat–Pea Intercrops
Source: Plants (Basel). 2021 Sep 23;10(10):1991. doi: 10.3390/plants10101991 (PMC8540429; doi:10.3390/plants10101991)
Supplement: Supplementary file 1 [file plants-10-01991-s001.zip › plants-1385237-supplementary.pdf]

Table S1. Effect of pea intercropping on the apparent root electrical capacitance (C_R*) of wheat at different plant ages (DAP: days after planting). Unpaired *t* test (n = 30) or Welch's test [superscript (W) in the table] was used, based on the homogeneity of variances. NS: non-significant; *p < 0.05; **p < 0.01; ***p < 0.001. Treatment codes: N: wheat cv. Mv Nádor; K: cv. Mv Kolompos; C: YQCCP population; 0: wheat sole crop; P: wheat–pea intercrop; (+): well-watered; (–): drought-stressed.

DAP	Groups compared					
	Well-watered			Drought-stressed		
	N0(+) vs. NP(+)	K0(+) vs. KP(+)	C0(+) vs. CP(+)	N0(–) vs. NP(–)	K0(–) vs. KP(–)	C0(–) vs. CP(–)
10	NS	NS	NS	NS	NS	NS
21	*	NS	NS	NS	NS	NS
31	*	*	*	**	***	NS
42	***	***	*	***	***	**
53	***	***	***	***	**	**
65	***	***	***	***	**	***
77	***	***	*	NS	***	***
88	***	***(W)	*	NS	*	**
101	NS(W)	*	NS	NS	NS	NS
115	NS	NS	NS	NS	NS	NS
129			NS			

Table S2. Effect of drought stress on the apparent root electrical capacitance (C_R*) of wheat at different plant ages (DAP: days after planting). Unpaired *t* test (n = 30) or Welch's test [superscript (W) in the table] was used, based on the homogeneity of variances. NS: non-significant; *p < 0.05; **p < 0.01; ***p < 0.001. For treatments codes, see Table S1.

DAP	Compared groups					
	N0(+) vs. N0(–)	NP(+) vs. NP(–)	K0(+) vs. K0(–)	KP(+) vs. KP(–)	C0(+) vs. C0(–)	CP(+) vs. CP(–)
10	NS	NS	NS	NS	NS	NS
21	***	***	NS	*	*	**
31	***	***	**	***	***	***
42	***(W)	***	***	***	***	***
53	***	***	***	***	***	***
65	***	***	***	***	***	***(W)
77	***	***	***	***(W)	***(W)	***(W)
88	***	***	***(W)	***	***(W)	***(W)
101	***(W)	***(W)	***(W)	***(W)	***(W)	***(W)
115	*	NS	***(W)	***(W)	***(W)	***(W)

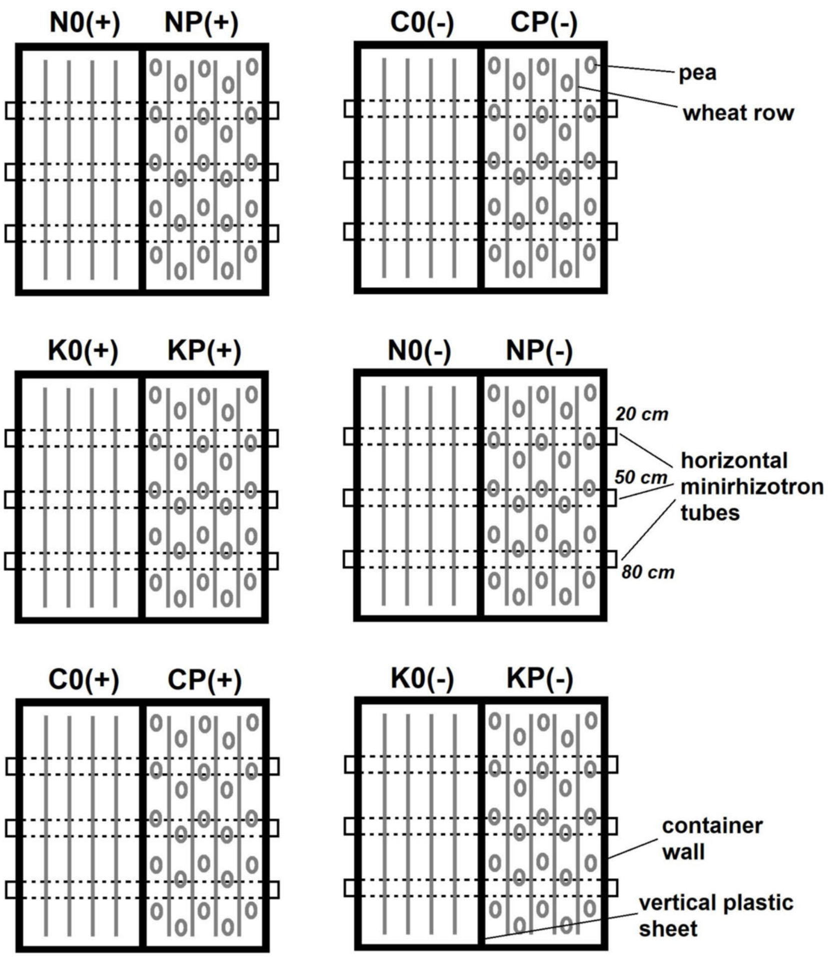

Figure S1. Schematic for the experimental design. Treatment codes: N: wheat cv. Mv Nádor; K: cv. Mv Kolompos; C: YQCCP population; 0: wheat sole crop; P: wheat–pea intercrop; (+): well-watered; (–): drought-stressed.

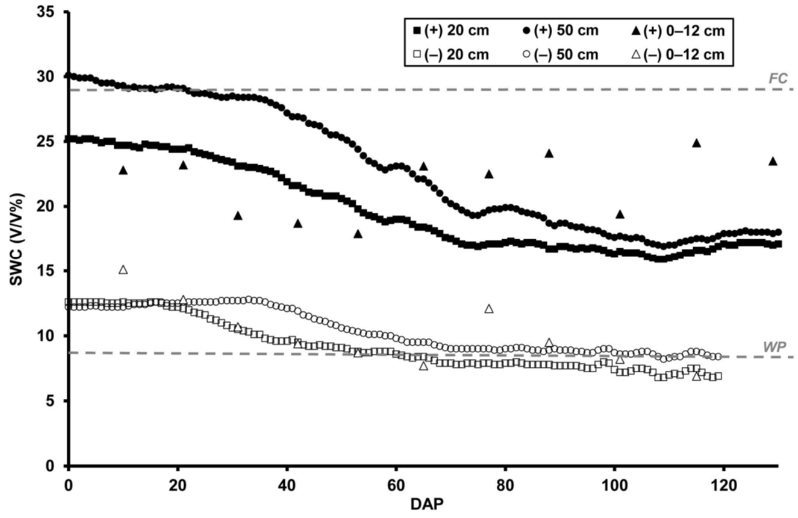

Figure S2. Changes in volumetric soil water content (SWC) with plant age (DAP: days after planting) in well-watered [(+)] and drought-stressed [(–)] treatments at different soil depths. SWC was recorded continuously at 20 and 50 cm depths using automatic data loggers, but only concurrently with root electrical capacitance measurements in the 0–12 cm layer with a handheld TDR meter. Data were averaged across the three wheat cultivars and two pea treatments. Horizontal dashed lines indicate water content at field capacity (FC; 0.02 MPa) and wilting point (WP; 1.5 MPa).
